# Impact of media compositions and culture systems on the immunophenotypes of patient-derived breast cancer cells

**DOI:** 10.1186/s12885-023-11185-7

**Published:** 2023-09-06

**Authors:** Seungyeon Ryu, So-Hyun Yoon, Junhyuk Song, Yoonjung Choi, Sangeun Lee, Moonjou Baek, Han-Byoel Lee, Sook Young Jeon, Sangyong Jon, Daeyoup Lee, Hoe Suk Kim, Wonshik Han

**Affiliations:** 1 Cancer Research Institute, Seoul National University, 101 Daehak-ro, Jongno-gu, Seoul, 03080 Republic of Korea; 2 Biomedical Research Institute, Seoul National University Hospital, 101 Daehak-ro, Jongno-gu, 03080 Seoul, Republic of Korea; 3 Interdisciplinary Programs in Cancer Biology Major, Seoul National University Graduate School, 103, Daehak-ro, Jongno-gu, 03080 Seoul, Republic of Korea; 4 Integrated Major in Innovative Medical Science, Seoul National University Graduate School, 103, Daehak-ro, Jongno-gu, 03080 Seoul, Republic of Korea; 5 Department of Biological Sciences, Korea Advanced Institute of Science and Technology, 291, Daehak-ro, Yuseong-gu, 34141 Daejeon, Republic of Korea; 6 Department of Surgery, Seoul National University College of Medicine, 103, Daehak-ro, Jongno-gu, Seoul, 03080 Republic of Korea; 7 Department of Surgery, Kangnam Sacred Heart Hospital, 1 Shingil-ro, Youngdeungpo-ku, 07441 Seoul, Republic of Korea; 8 Department of Pharmaceutical Sciences, College of Pharmacy & Allied Health Professions, South Dakota State University, SAV# 255, Box2202C, Brookings, SD 57007 USA; 9 Genomic Medicine Institute, Medical Research Center, Seoul National University, 103, Daehak- ro, Jongno-gu, 03080 Seoul, Republic of Korea

**Keywords:** Patient-derived breast cancer cell, Immunophenotype, Breast cancer stem cell, Progenitor, Estrogen receptor, Monolayer, Spheroid

## Abstract

**Background:**

Heterogeneous tumor cells are thought to be a significant factor in the failure of endocrine therapy in estrogen receptor-positive (ER+) cancers. Culturing patient-derived breast cancer cells (PDBCCs) provides an invaluable tool in pre-clinical and translational research for the heterogeneity of cancer cells. This study aimed to investigate the effects of different media components and culture methods on the BCSC-associated immunophenotypes and gene expression in ER + PDBCCs.

**Methods:**

Ten patients with ER + breast cancer were employed in this study, six of whom had neoadjuvant chemotherapy and four of whom did not. PDBCCs were isolated by enzymatic methods using collagen I and hyaluronidase. PDBCCs were grown as monolayers in mediums with different compositions and as multicellular spheroid in a suspended condition. Collagen I-coated plate and ultralow attachment plate coated with polymer-X were used for monolayer and spheroid culture. Flow cytometry, immunofluorescent staining, RT-PCR, and RNA-sequencing were employed to examine the immunophenotype and genetic profile of PDBCCs.

**Results:**

More than 95% of PDBCCs sustain EpCAM high/+/fibroblast marker- phenotypes in monolayer conditions by subculturing 3–4 times. A83-01 removal induced senescent cells with high β-galactosidase activity. PDBCCs grown as monolayers were characterized by the majority of cells having an EpCAM+/CD49f + phenotype. Compared to full media in monolayer culture, EGF removal increased EpCAM+/CD49f − phenotype (13.8-fold, p = 0.028), whereas R-spondin removal reduced it (0.8-fold, p = 0.02). A83-01 removal increased EpCAM+/CD24 + phenotype (1.82-fold, p = 0.023) and decreased EpCAM low/-/CD44+/CD24- phenotype (0.45-fold, p = 0.026). Compared to monolayer, spheroid resulted in a significant increase in the population with EpCAM-/CD49+ (14.6-fold, p = 0.006) and EpCAM low/-/CD44+/CD24- phenotypes (4.16-fold, p = 0.022) and ALDH high activity (9.66-fold, p = 0.037). ALDH1A and EMT-related genes were upregulated. In RNA-sequencing analysis between spheroids and monolayers, a total of 561 differentially expressed genes (2-fold change, p < 0.05) were enriched in 27 KEGG pathways including signaling pathways regulating pluripotency of stem cells. In a recurrence-free survival analysis based on the Kaplan-Meier Plotter database of the up-and down-regulated genes identified in spheroids, 15 up-, and 14 down-regulated genes were associated with poor prognosis of breast cancer patients.

**Conclusion:**

The media composition and spheroid culture method change in the BCSCs and EMT markers of PDBCCs, implying the importance of defining the media composition and culture method for studying PDBCCs in vitro.

**Supplementary Information:**

The online version contains supplementary material available at 10.1186/s12885-023-11185-7.

## Introduction

Endocrine therapy is the most effective treatment for patients with estrogen receptor-positive (ER+) breast cancer. However, 20–25% of patients with the ER + subtype did not respond clinically to endocrine therapy [1–3]. Progenitors and breast cancer stem cells (BCSCs), which contribute to the intratumor heterogeneity of ER + breast cancer, may be a major culprit in the failure of endocrine therapy [4–7]. Analyzing the genetic and cellular characteristics of the breast cancer cell lineage is essential to understanding the heterogeneity of breast cancer.

Immunophenotyping on the surface of breast cancer cells approached by flow cytometry can be a useful tool to reveal the molecular characteristics of individual cells [8]. Breast cancer cells in tumor tissues exhibit luminal, basal, and BCSCs phenotypes based on the expression of epithelial cell adhesion molecule (EpCAM), CD49f (α6 integrin), CD44, and CD24; EpCAM+/CD49f- mature luminal cells, EpCAM+/CD49f + luminal progenitors, EpCAM-/CD49f + basal progenitors [9, 10], BCSCs with EpCAM low/-/CD44+/CD24- marker and high activity of aldehyde dehydrogenase 1 (ALDH1) [6, 7].

Primary culture of patient-derived breast cancer cells (PDBCCs) has been extensively used to reflect the breast cancer cell characteristics in patient tumors. Many studies have explored how the proper medium composition and specialized culture method impact PDBCCs’ properties over an extended length of time in vitro [11–13]. Three-dimensional (3D) multicellular spheroids culture is a method for mimicking the in vivo biological behavior of breast cancer cells and enriching BCSCs [14, 15]. The polymer-X thin film platform allows for the easy generation of tumorigenic 3D spheroids from diverse cancer cell lines [16, 17].

In this study, PDBCCs were extracted from tumor tissues of ER + breast cancer patients and grown in different composition media as 2D adherent monolayers on collagen I-coated plates and 3D spheroids utilizing a polymer-X thin film platform. We investigated the effects of different media components and culture methods on the immunophenotypes and gene expression associated with BCSC in ER + PDBCCs.

## Methods

### Patients and tumor tissues

Fresh breast tumor samples were collected from ten ER + breast cancer patients treated at Seoul National University Hospital with the agreement of the Ethics Committee. The Seoul National University Hospital’s Institutional Review Board approved this study (H-2007-204-1145). All experiments were carried out with each subject’s understanding and written agreement. This research was carried out in line with the Helsinki Declaration.

### Breast cancer cell isolation and monolayer culture

Surgical tumor tissues of ER + patients were digested in 10ml advanced DMEM/F12 (Invitrogen, Waltham, MA, USA) containing 10 mM HEPES (Gibco, Waltham, MA, USA), and antibiotics (Gibco, Waltham, MA, USA), for 3 h with a mixture of 1ml collagenase/hyaluronidase (STEMCELL Technologies, Vancouver, BC, Canada) and 300 µg/ml DNase I (Roche, Basel, Switzerland), then strained through a 100 μm filter and centrifuged at 500 g for 5 min. PDBCC monolayers were produced and propagated on collagen I-coated plates using modified organoid medium composition (with 0.16 nM estradiol and without P38 inhibitor) described by Sachs et al. [13].

### 3D multicellular spheroid formation on polymer-X

PDBCCs (1 × 10^6^) were seeded on polymer-X coated 6 well plates (KAIST, Daejeon, Korea). For flow cytometry and real-time RT-PCR, PDBCCs were grown in the modified organoid medium composition containing 0.16 nM estradiol but not P38 inhibitor [13]. For RNA-seq, PDBCCs were grown in serum-free F12-dulbecco’s modified Eagle’s medium (Sigma, Burlington, MA, USA) supplemented with 20 ng/ml EGF (Invitrogen, Waltham, MA, USA), 20 ng/ml bFGF (Millipore, Burlington, MA, USA), 10 ng/ml LIF (Millipore, Burlington, MA, USA), B27 supplement (Invitrogen, Waltham, MA, USA), and antibiotic-antimycotic (Invitrogen, Waltham, MA, USA), which represented as NDY media and mTeSR media provided by STEMCELL Technologies at 37 °C in a humidified 5% CO2 atmosphere. The culture media was changed every 2 to 3 days to ensure optimal spheroid growth. PDBCC spheroids were generated by suspension culture for 7–8 days.

### Flow cytometry

Single cells dissociated from spheroids or monolayers with TrypLE™ (Thermo Fischer, Waltham, MA, USA) were stained using concentrations of fluorochrome-conjugated monoclonal antibodies recommended by the manufacturer for 20 min at room temperature in the dark. Antibodies for EpCAM-APC (BioLegends, San Diego, California, USA), CD24-FTIC (BioLegends, San Diego, California, USA), CD44-PE (BioLegends, San Diego, California, USA), CD49f-FITC (BioLegends, San Diego, California, USA), and fibroblast-marker-FITC (BioLegends, San Diego, California, USA) were used. Expression of EpCAM, CD24, CD44, CD49f, and fibroblast-marker on PDBCCs was analyzed by multicolor flow cytometry on a FACS Canto II flow cytometer (BD Biosciences, Becton Drive Franklin Lakes, NJ, USA). The gating strategy was provided in Supplementary Fig. 1.

### Senescence-associated-β-galactosidase (SA-β-gal) staining

SA-β-gal staining was conducted on monolayer cells using a Cellular Senescence Assay Kit (Cell Biolabs Inc., San Diego, CA, USA) according to the manufacturer’s protocol. Cells stained for SA-β-gal were imaged using a camera mounted on a light microscope (Olympus Corporation, Tokyo, Japan). Areas measuring 0.44 mm × 0.32 mm (length × width) from each image were scanned. The results of “% area” were used as the stained level of SA-β-gal.

### Immunofluorescence staining

Monolayers and spheroids were washed in PBS before being fixed for 30 min at 4 °C with 2% paraformaldehyde, followed by blocking with 2% bovine serum albumin. Cells were reacted with primary antibodies for EpCAM (Sigma, Burlington, MA, USA), Pancytokeratin (Sigma, Burlington, MA, USA), and CK8/18/19 (Sigma, Burlington, MA, USA) and secondary antibodies conjugated to Alexa 594 and 488 (Invitrogen, Waltham, MA, USA) and were counterstained with 4’,6-diamidino-2-phenylindole for nucleus. The cell images were acquired using a confocal microscope (Leica, Wetzlar, Germany).

### ALDEFLUOR assay

The ALDH activity of cells was determined using the ALDEFLUOR reagent system (STEMCELL Technologies, Vancouver, BC, Canada) according to the manufacturer’s procedure. Cells dissociated from spheroids were suspended in ALDEFLUOR™ assay buffer with a BODIPY-aminoacetaldehyde diethyl acetal (BAAA-DA), for 40 min at 37 °C. The negative control was given 50 µmol/L of diethylaminobenzaldehyde (DEAB), an ALDH-specific inhibitor. Cells with high ALDH activity were examined using a FACS Canto II flow cytometer (BD Biosciences, Becton Drive Franklin Lakes, NJ, USA).

### Real-time RT-PCR analysis

Total RNA was extracted from cultured cells using TRIzol Reagent (Takara, Kusatsu, Shiga Japan). cDNA was produced using a cDNA kit (Applied Biosystems, Foster City, CA, USA). The specific primers for ALDH1A1, ALDH1A2, ALDH1A3, E-cadherin, N-cadherin, ZEB1 and 2, Snail, Slug, Twist, fibronectin, vimentin, matrix metallopeptidase 9 (MMP9), and glyceraldehyde-3-phosphate dehydrogenase (GAPDH) were shown in Table 1. Real-time PCR reactions were run on an ABI PRISM® 7900 using a SYBR Green PCR master mix (Applied Biosystems, Foster City, CA, USA). The results were analyzed by the ∆∆Ct method, which reflects the difference in the threshold for the target gene relative to that of GAPDH in each sample. Gene levels of spheroids were assessed in comparison to those of their monolayer counterparts.

**Table 1:** Specific primer sequences used for real-time RT-PCR

Gene	Sequence (5′$->$3′)	
E-cadherin	Forward	ATTCTGATTCTGCTGCTCTTG
	Reverse	AGTAGTCATAGTCCTGGTCTT
N-cadherin	Forward	CTCCTATGAGTGGAACAGGAACG
	Reverse	TTGGATCAATGTCATAATCAAGTGCTGTA
ZEB1	Forward	TTCAAACCCATAGTGGTTGCT
	Reverse	TGGGAGATACCAAACCAACTG
ZEB2	Forward	CAAGAGGCGCAAACAAG
	Reverse	GGTTGGCAATACCGTCATCC
Snail	Forward	GAGGCGGTGGCAGACTAG
	Reverse	GACACATCGGTCAGACCAG
Slug	Forward	CATGCCTGTCATACCACAAC
	Reverse	GGTGTCAGATGGAGGAGGG
Twist	Forward	CGGGAGTCCGCAGTCTTA
	Reverse	TGAATCTTGCTCAGCTTGTC
Fibronectin	Forward	CAGAATCCAAGCGGAGAGAG
	Reverse	CATCCTCAGGGCTCGAGTAG
Vimentin	Forward	CCCTCACCTGTGAAGTGGAT
	Reverse	TCCAGCAGCTTCCTGTAGGT
MMP9	Forward	CAACATCACCTATTGGATCC
	Reverse	CGGGTGTAGAGTCTCTCGCT
CD24	Forward	TCTAAATGTGGCTATTCTGATCCA
	Reverse	TATTTGGGAAGTGAAGACTGGAA
CD44	Forward	TCCAACACCTCCCAGTATGACA
	Reverse	GGCAGGTCTGTGACTGATGTACA
ALDH1A1	Forward	TGTTAGCTGATGCCGACTTG
	Reverse	TTCTTAGCCCGCTCAACACT
ALDH1A2	Forward	CTGGCAATAGTTCGGCTCTCTC
	Reverse	TGATCCTGCAAACATGCTC
ALDH1A3	Forward	TCTCGACAAAGCCCTGAAGT
	Reverse	TATTCGGCCAAAGCGTATTC
GAPDH	Forward	GCACCGTCAAGGCTGAGAAC
	Reverse	TGGTGAAGACGCCAGTGGA

### RNA-Seq analysis

mRNA was extracted from PDBCCs spheroids cultured for 8 days on pV4D4-coated plates and monolayer cultured PDBCCs using a Magnetic mRNA Isolation Kit (NEB, Ipswich, MA, USA) according to the manufacturer’s protocol. A library was prepared from DNase-treated mRNA using a NEBNext® Ultra™ II RNA Library Prep Kit for Illumina®, as described by the manufacturer. Each library was sequenced on a Novaseq6000 system using the paired-end method (150-bp reads). The sequenced reads were aligned to the human genome (version: Hg19) using a STAR aligner (v.2.4.0). The HOMER software algorithm [18] and DESeq R package were used to investigate differentially expressed genes (DEGs) between spheroids and monolayers. Heatmap and MA plots were visualized using the heatmap function and plotMA function, respectively, of the R statistical programming language v.3.3.0. (http://www.r-project.org/). P-values of 0.05 and log ratios of 2 were used to identify genes that were upregulated and downregulated.

### Kyoto Encyclopedia of genes and genomes (KEGG) pathway and enrichment analysis

To gain insight into the underlying biology of DEGs related to SerpinB2 loss, biological functional categories enriched in the DEGs were identified using the functional annotation and clustering tool of the Database for Annotation, Visualization, and Integrated Discovery v6.7 (https://david.ncifcrf.gov/) [26–28]. To further understand the biological functions of the DEGs, KEGG pathway enrichment analyses were performed using the DAVID online tool. P < 0.05 was set as the threshold value.

### Kaplan–Meier (KM) plotter analysis

The Kaplan-Meier Plotter (http://www.kmplot.com/analysis) examined public microarray data repositories for DEGs discovered between spheroids and monolayers on relapse-free survival (RFS) of ER + breast cancer patients (N = 3499) with a mean follow-up of 240 months. The median threshold value for DEGs expression was selected, dividing the patient samples into two groups, and graphs were constructed accordingly.

### Statistical analyses

All data were presented as the mean ± standard deviation of at least three independent experiments. Paired or unpaired t-tests and Mann-Whitney U test were used to make statistical comparisons between the two independent groups. For groups of three or more, data were analyzed using one-way ANOVA, followed by Dunnett’s multiple comparison test. GraphPad Prism v9.2.0 was used for statistical analysis (GraphPad Software Inc., La Jolla, CA, USA). P values of less than 0.05 were considered statistically significant.

## Results

### Patient clinical data

Detailed clinical, pathologic, treatment, and follow-up data of 10 patients with ER + breast cancer obtained from patients’ medical records were shown in Table 2. The age of patients ranged from 36 to 62 years, and the median age was 44.7 years. All patients received hormone therapy. Five patients received both neoadjuvant chemotherapy and adjuvant chemotherapy. Out of 10 cases, the most frequent histological subtype was invasive ductal carcinoma (IDC) in 9 cases, of which 1 showed mucinous carcinoma. The tumor size was < 2 cm (pT1) in 2 cases, between 2 and 5 cm (pT2) in 6 cases, and > 5 cm (pT4) in 2 cases. 8 cases had axillary lymph node (LN) involvement, and 1 case (#158) had lung metastasis. As shown in Table 2, patients received risk-adapted neoadjuvant and adjuvant chemotherapy with surgery, with or without radiotherapy, to control primary and possibly metastatic disease. Patient (#158) with lung metastasis was chosen to stop cancer treatment and focused on palliative care. On findings confirmed by IHC, 9 cases expressed ER strongly (80–90%). Both ER and PR were positive in 6 cases. Of the 3 patients with a score of 2 + by HER-2 IHC, 1 case (#110) was FISH positive for HER-2 gene amplification and classified as luminal B subtype. On the evaluation of the intrinsic subtype using the tumor proliferation index marker Ki-67 in our hospital, the Ki-67 index of tumors with luminal B type was > 10%. 3 cases (#106, #107, and #158) were luminal B type, which ranged in Ki-67 index from 17 to 70%. A high Ki-67 index (70%) in 1 case (#158) correlated with a greater risk of distance metastasis.

**Table 2:** Clinical parameters of breast cancer patients with ER + subtype.

Case No	Age	Intrinsic subtype	Dx	NCT regimen	CTx regimen	HTx	RT	Pathology stage	Metastatic site	Surgical specimen IHC							
										NG	HG	ER (%)	PR (%)	HER2	AR (%)	P53 (%)	Ki67 (%)
#69	36	LumA	IDC	AC ->D	AC ->P	zoladex + TMX	1	T2N1M0		3	2	80	0	++	50	< 25	1
#106	54	LumB	IDC	AC	T	arimidex		T4N1M0		3	3	2	0	-	0	> 75	17
#107	42	LumB	IDC		AC ->P	TMX		T1N1M0		3	2	90	30	-	90	25–50	50
#108	37	LumA	Mucinous			TMX + leuplin		T2N0M0		2	2	90	80	-	90	25–50	7
#110	40	LumB	IDC	AC	DH	TMX		T2N1M0		3	3	80	10	++	90	> 75	1
#132	37	LumA	IDC	FAC	TC	letrozole + leuplin		T2N1M0		3	3	90	3	-	90	< 25	3
#158	50	LumB	IDC	AC->D		zoladex + TMX		T4N2M1	lung	3	2	90	0	+	50	> 75	70
#168	44	LumA	IDC		AC ->D	TMX	1	T2N1M0		2	2	90	90	-	90	50–75	3
#170	62	LumA	IDC	AC -> D	xeloda	arimidex	1	T2N1M0		3	2	90	0	+	5	< 25	1
#190	41	LumA	IDC			letrozole		T1N1M0		2	2	90	30	++	90	a few	1

IHC, immunohistochemistry; Dx, disease type; IDC, invasive ductal carcinomas; NG, nuclear grade; HG, histologic grade; ER, estrogen receptor; PR, progesterone receptor; HER2, human epidermal receptor 2; AR, androgen receptor; LumA, luminal A; LumB, luminal B; NCT, neoadjuvant chemotherapy; CTx, adjuvant chemotherapy; HTx, hormone therapy; RT, radiotherapy; AC, doxorubicin + cyclophosphamide; D, docetaxel; P, paclitaxel; T, taxen; DH, docetaxel + traztumab; FAC, 5-fluorouracil + doxorubicin + cyclophosphamide; TMX, tamoxifen

### EGF, R-spondin, and ALK modulate the immunophenotype of PDBCCs grown as monolayer

**Fig. 1:**
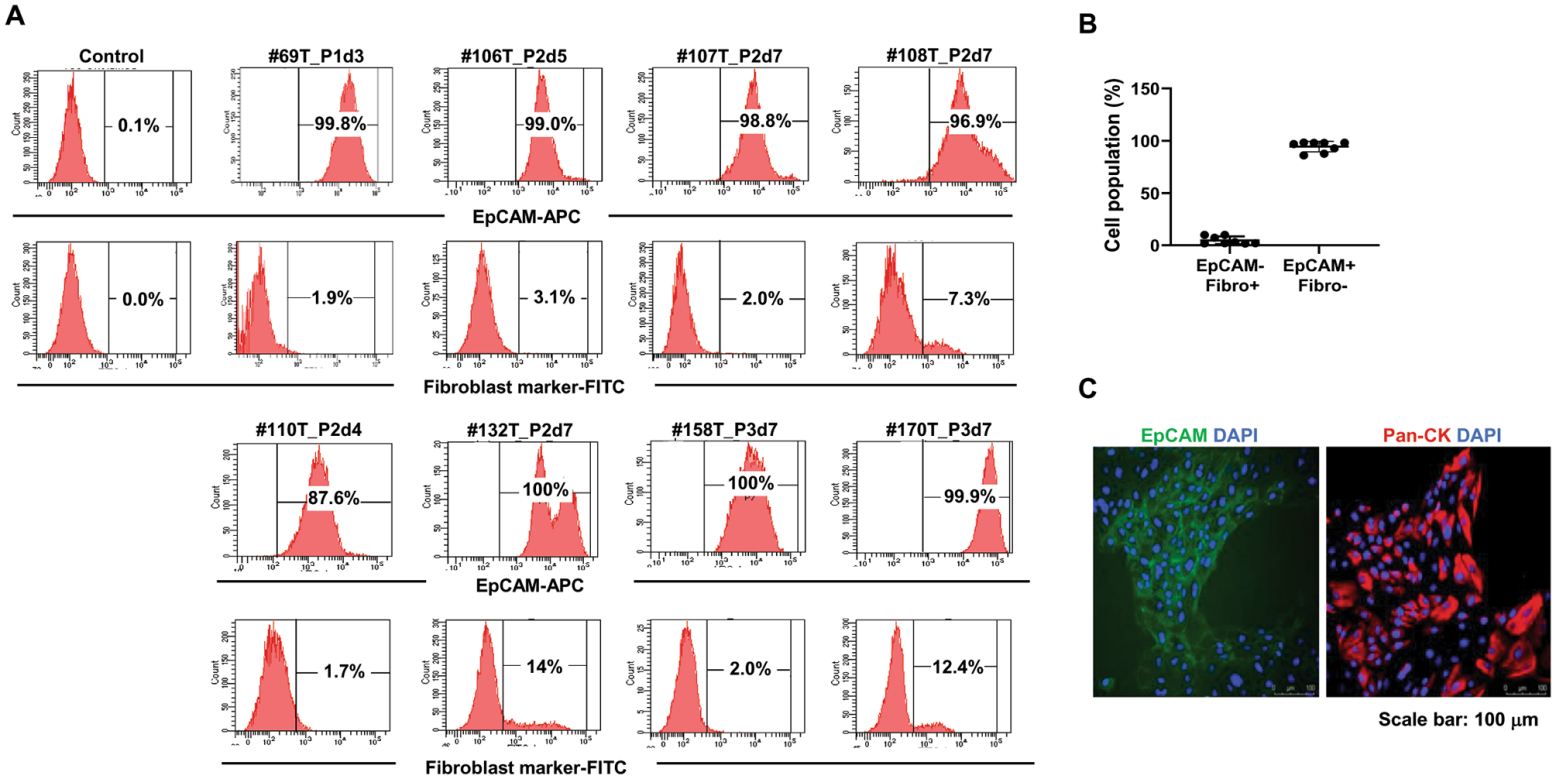
Flow cytometry analysis of EpCAM and fibroblast marker in PDBCCs grown as monolayer on a collagen I-coated plate. (**A**) Representative flow cytometric histogram plot and (**B**) Quantification (%) of EpCAM + and fibroblast marker- cell population in PDBCCs obtained from 8 patients. Data represent the mean ± standard deviation (n=8). (**C**) Representative immunofluorescent staining for EpCAM and pancytokeratin in PDBCCs. Scale bar: 100 μm

EpCAM is a tumor-associated antigen that has been identified as a marker for most epithelial malignancies, including breast cancer. PDBCCs were extracted from fresh surgical tumor tissues and cultured for 7–10 days in Matrigel on a culture plate before being coupled to collagen I-coated plates and flow cytometry analysis to identify EpCAM-positive breast cancer cells and fibroblast marker-positive cancer-associated fibroblasts. EpCAM-positive cells separated from each tumor tissue vary from 88 to 100%, and fibroblast marker-positive cells were less than 15%, as illustrated in Fig. 1A. After 2–3 times of subculture, 94.48 ± 5.0% of the PDBCCs obtained from 10 breast cancer patients had EpCAM-positive and fibroblast marker-negative (EpCAM+/Fibro-) (Fig. 1B). Immunostaining revealed that most of PDBCCs highly expressed EpCAM as well as cytokeratin, specific markers of epithelial cancer cells (Fig. 1C).

**Fig. 2:**
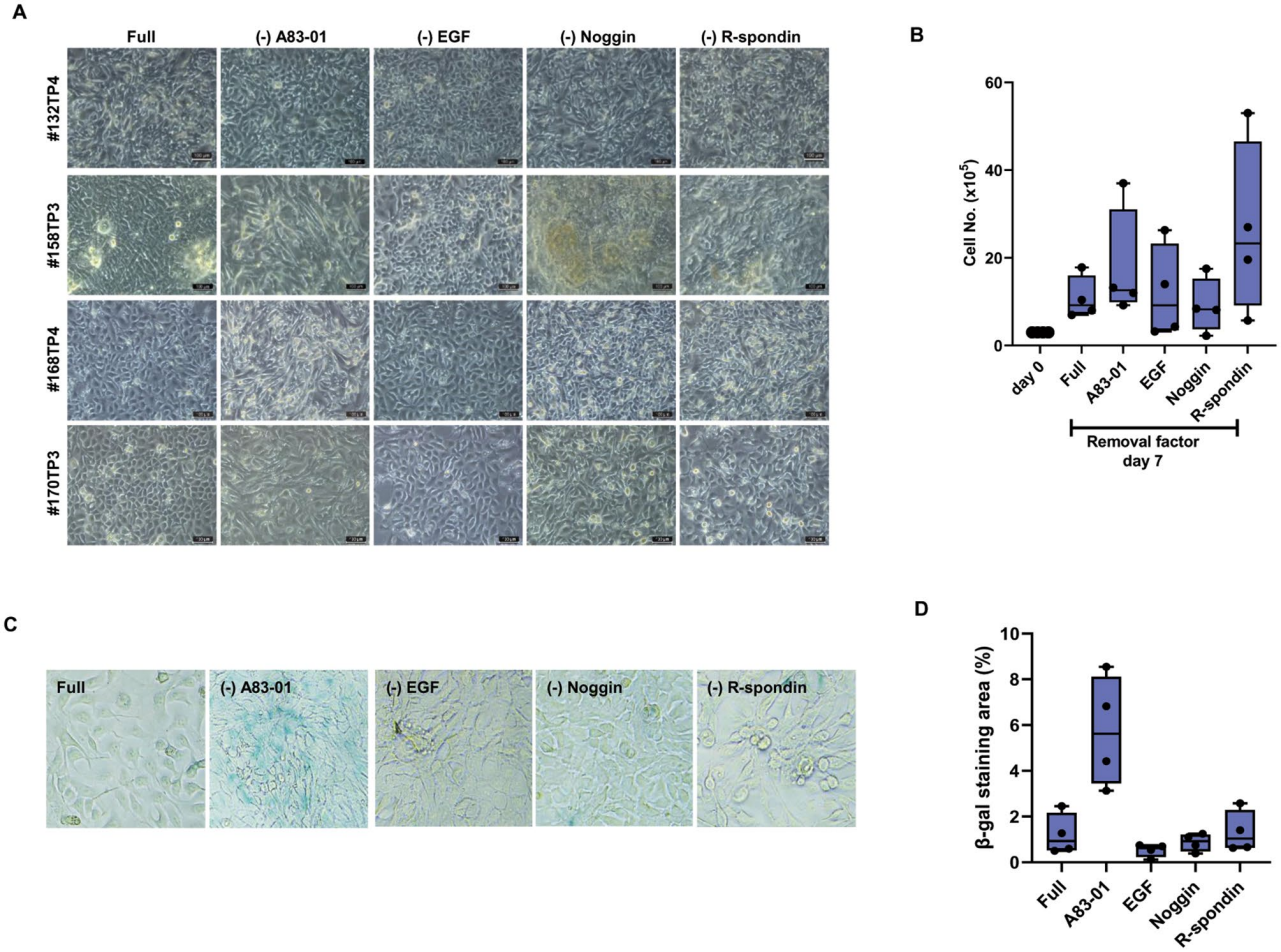
Effect of media factors on growth and senescence of PDBCCs grown as monolayer. Analysis of growth rate and senescence in PDBCCs grown as monolayer by removals of A83-01 (500 nM), EGF (5 ng/ml), Noggin (100 ng/ml), and R-spondin (250 ng/ml). (**A**) Representative images and (**B**) Quantitative growth analysis of PDBCCs cultivated for 7 days. Data represent the mean ± standard deviation (n=4). (**C**) Representative images and (**D**) Quantitative analysis of senescence-associated β-galactosidase (SA-β-gal) staining of PDBCCs cultivated for 7 days. Data represent the mean ± standard deviation (n=4)

In this study, PDBCCs were grown in full media as reported by Sachs N et al. [13]. To determine the contribution of the individual growth factors and inhibitors to the representation of different mammary epithelial cell types in monolayer culture, PDBCCs were cultivated in the media without the epidermal growth factor (EGF), the Wnt-agonist R-spondin, the transforming growth factor beta (TGF-β) inhibitor Noggin, the activin receptor-like kinase (ALK) inhibitor A83-01 for 7 days. Even the removal of EGF, A83-01, Noggin, or R-spondin did not lead to a significant alteration in growth rate, the microscopic morphology of PDBCCs was altered. PDBCCs obtained from 4 patients had a cuboidal and polygonal morphology in full media. PDBCCs with an elongated and fibroblast-like form emerged in media lacking A83-01, Noggin, and R-spondin for 7 days; however, their number decreased when EGF was removed (Fig. 2A-B).

The primary cells have a limited lifespan and reach replicative senescence after a certain number of cell divisions. To investigate if media components contribute to reaching replicative senescence, the senescent states of PDBCCs have been assessed by comparing the staining areas of senescence-associated beta-galactosidase (SA-β-gal), which is used as the biomarker for senescent cells. Even while there is no appreciable difference in the area stained with β-gal when compared to the whole media, the removal of A83-01 increased the SA-β-gal-positive stained areas (Fig. 2C-D).

**Fig. 3:**
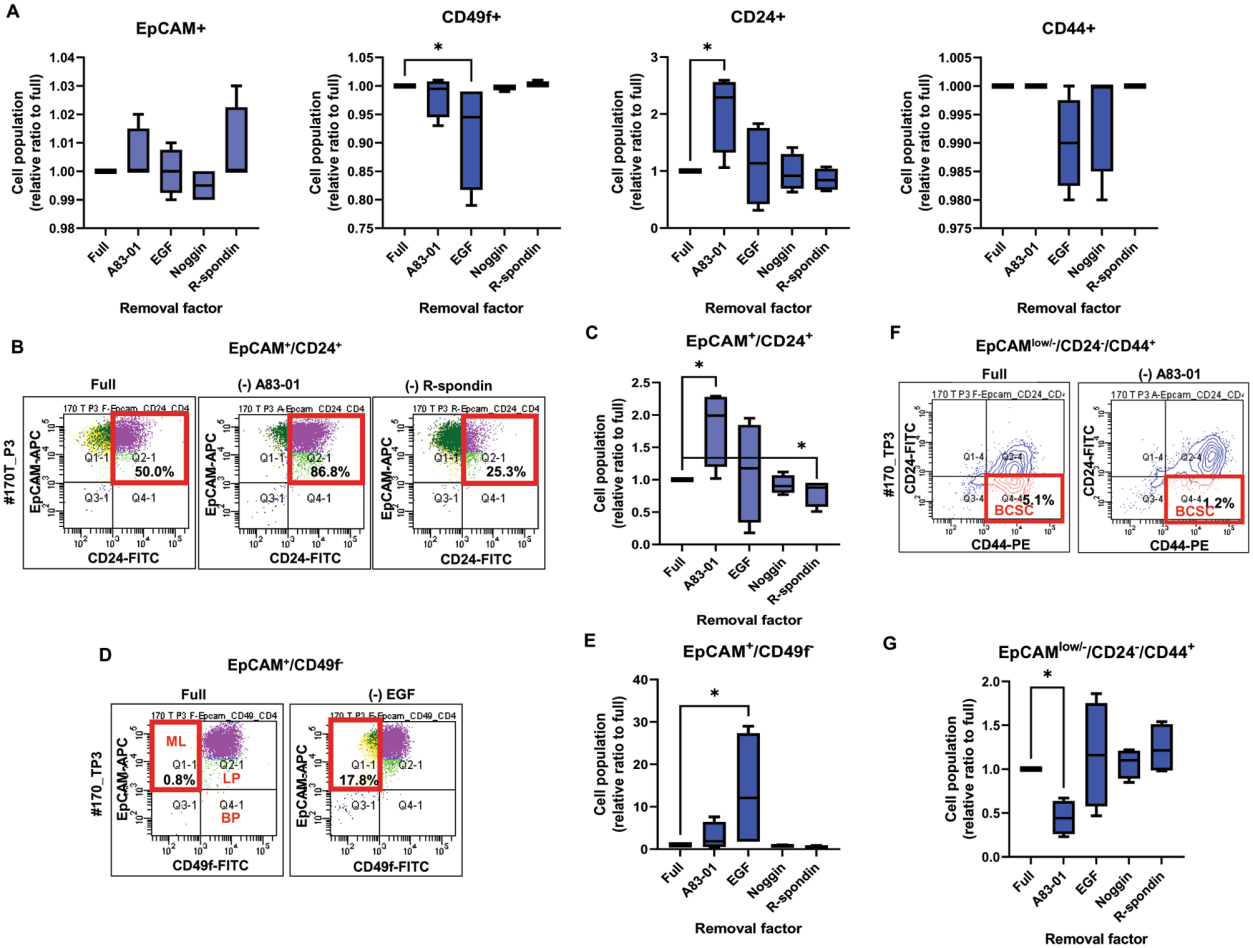
Effect of media factors on immunophenotypes of PDBCCs grown as monolayer. Flow cytometry analysis of single and multiple stained PDBCCs grown as monolayer by removals of A83-01 (500 nM), EGF (5 ng/ml), Noggin (100 ng/ml), and R-spondin (250 ng/ml). (**A**) Flow cytometry quantification of EpCAM-, CD49f-, CD24-, CD44-signle stained cells. (**B**) Representative dot plots and (**C**) Quantification of EpCAM- and CD24-double stained cells. (**D**) Representative dot plots and (**E**) Quantification of EpCAM- and CD49f-double stained cells (**F**) Representative dot plots and (**G**) Quantification of EpCAM-, CD24-, and CD44-triple stained cells cultivated in media without A83-01, EGF, Noggin, and R-spondin as compared to full media. Data represent the mean ± standard deviation (n=4). *p < 0.05 as compared to Full using one way-ANOVA followed by Dunnett’s multiple comparison test.

The immunophenotypes of PDBCCs were analyzed using flow cytometry. As compared with full media, EGF removal resulted in a significant decrease in a cell population with CD49f as a marker of epithelial stem cells (0.9-fold, p = 0.028) and A83-01 removal increased cell population with CD24 (anchored cell surface glycoprotein) (2.06-fold, p = 0.028) (Fig. 3A). A83-01 removal enhanced EpCAM+/CD24 + cells (1.82-fold, p = 0.023) a marker of mature luminal epithelial phenotype (Fig. 3B and C), but decreased EpCAM low/-/CD24-/ CD44 + cells (0.45-fold, p = 0.026) BCSC marker (Fig. 3F and G); R-spondin removal decreased EpCAM+/CD24 + cells (0.8-fold, p = 0.023) (Fig. 3B and C); EGF removal increased EpCAM+/CD49f − cells (13.8-fold, p = 0.028), a marker of mature luminal phenotype (Fig. 3D and E).

### PDBCCs form cohesive spheroids on ultra-low attachment polymer-X platform

**Fig. 4:**
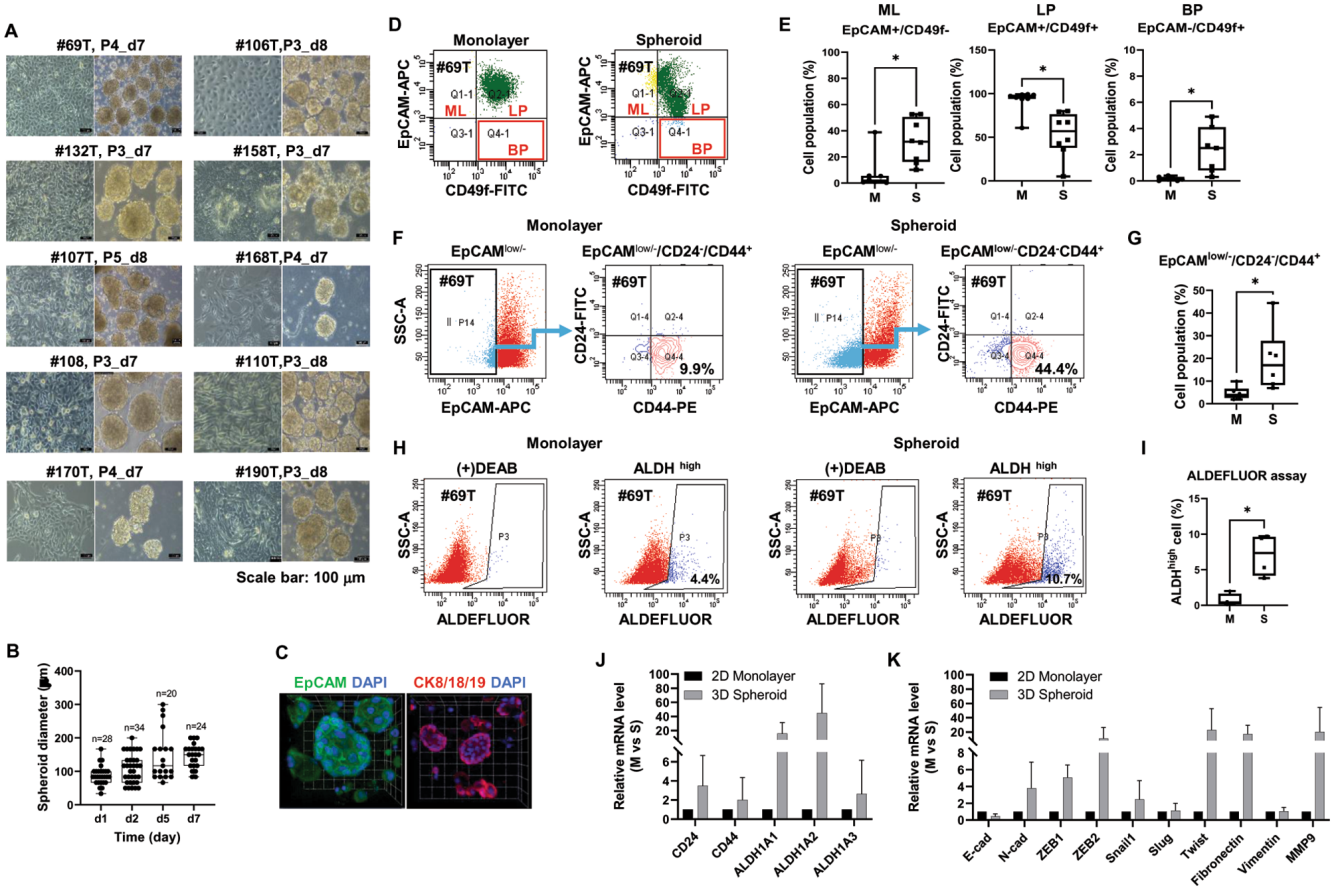
Comparison of immunophenotypes and gene expressions of PDBCCs grown as multicellular spheroids and monolayers in modified organoid medium using flow cytometry, ALDH activity, and qRT-PCR analyses (**A**) Representative monolayers and spheroids of 10 patients cultivated in full media for 7–8 days. (**B**) Quantitative spheroids diameters. Data represent the mean ± standard deviation (n=20-33). (**C**) Representative immunofluorescent staining for EpCAM and CK8/18/19 in spheroids. (**D**) Representative dot plots and (**E**) Quantification of EpCAM- and CD49f-double stained cells in monolayers and spheroids. Data represent the mean ± standard deviation (n=7-8). (**F**) Representative dot plots and (**G**) Quantification of EpCAM-, CD24, and CD44-triple stained cells in monolayers and spheroids. Data represent the mean ± standard deviation (n=6). (**H**) Representative dot plots and (**I**) Quantification of ALDEFUOR™ assays in monolayers and spheroids. Data represent the mean ± standard deviation (n=4). (**J**-**K**) qRT-PCR analysis in monolayers and spheroids. Data represent the mean ± standard deviation (n=3-4). *p < 0.05, **p < 0.01 as compared to monolayer (M) and spheroid (S) using t-test

During subculture up to passage 3–4, PDBCCs were grown as spheroids on polymer-X-coated plates (Fig. 4A). Although the size of the spheroids was different for each patient, the PDBCCs formed solid spherical multicellular aggregates within 1–2 days, and the maximum diameter of the spheroids reached 200 μm on day 7 of culture (Fig. 4B). Cytokeratin (CK) is used to examine luminal (CK 8, 18, and 19) phenotypes of invasive breast cancer. As we expected, PDBCCs grown as spheroids highly expressed EpCAM and CK8/18/19 (Fig. 4C).

### Spheroid culture leads to an increase in progenitors and BCSCs displaying the phenotypes of EpCAM-/CD49f+, EpCAM low/-/CD24-/CD44+, and high ALDH activity

To learn if the percentage of progenitors and BCSCs increases in spheroids, we analyzed luminal progenitor with EpCAM+/CD49f + phenotype, stem/basal progenitor with EpCAM−/CD49f + phenotype, BCSCs with EpCAM low/-/CD24-/CD44 + phenotype and BCSCs with high ALDH activity in monolayer and spheroid from the same patients using flow cytometry and the ALDEFLUOR™ assay. Figure 4 A showed the monolayer and multicellular spheroids images of 10 cases. The diameter of multicellular spheroids was increased up to about 200 µM at 7 days (Fig. 4B). Spheroids expressed EpCAM and CK8/18/19 strongly (Fig. 4C). The majority of PDBCCs cultured in monolayer and spheroids displayed EpCAM+/CD49f + cells (M vs. S; 91.76 ± 13.87% vs. 59.89 ± 17.78%, p = 0.002). The percentage of EpCAM-/CD49f + cells was significantly increased in spheroid (2.34 ± 1.73%) compared to monolayer (0.16 ± 0.15%) (p = 0.006, Fig. 4D-E). The percentage of EpCAM low/-/CD24-/CD44 + cells evaluated from total cells was found to be significantly higher in the spheroids (19.37 ± 13.80%) than in monolayers (4.65 ± 2.87%) (p = 0.022, Fig. 4F-G). The percentage of ALDH + cells detected by using the ALDEFLUOR™ assay was also significantly high in spheroids (7.05 ± 2.95%) relative to monolayers (0.73 ± 0.86%) (p = 0.037, Fig. 4H-I). In comparison to organoids, spheroids are significantly concentrated in basal progenitor EpCAM-/CD49 + and ALDH high BCSCs (Supplementary Fig. 2).

To further compare the expressions of BCSC-associated genes between spheroids and monolayer, the expressions of five BCSC markers (CD24, CD44, ALDH1A1, ALDH1A2, ALDH1A3) and six epithelial-mesenchymal transition (EMT)-associated genes (E-cadherin, N-cadherin, Snail1, Slug, Fibronectin, Vimentin) were analyzed by RT-qPCR. mRNA levels of CD44, CD24 and ALDH1A1-3 increased in spheroids in comparison to monolayers (Fig. 4J). mRNA levels of N-cadherin, Snail1, and Fibronectin were upregulated in spheroids, but E-cadherin mRNA was downregulated (Fig. 4K).

### Comparison of clinically poor prognosis-associated genes between monolayers and spheroids

**Fig. 5:**
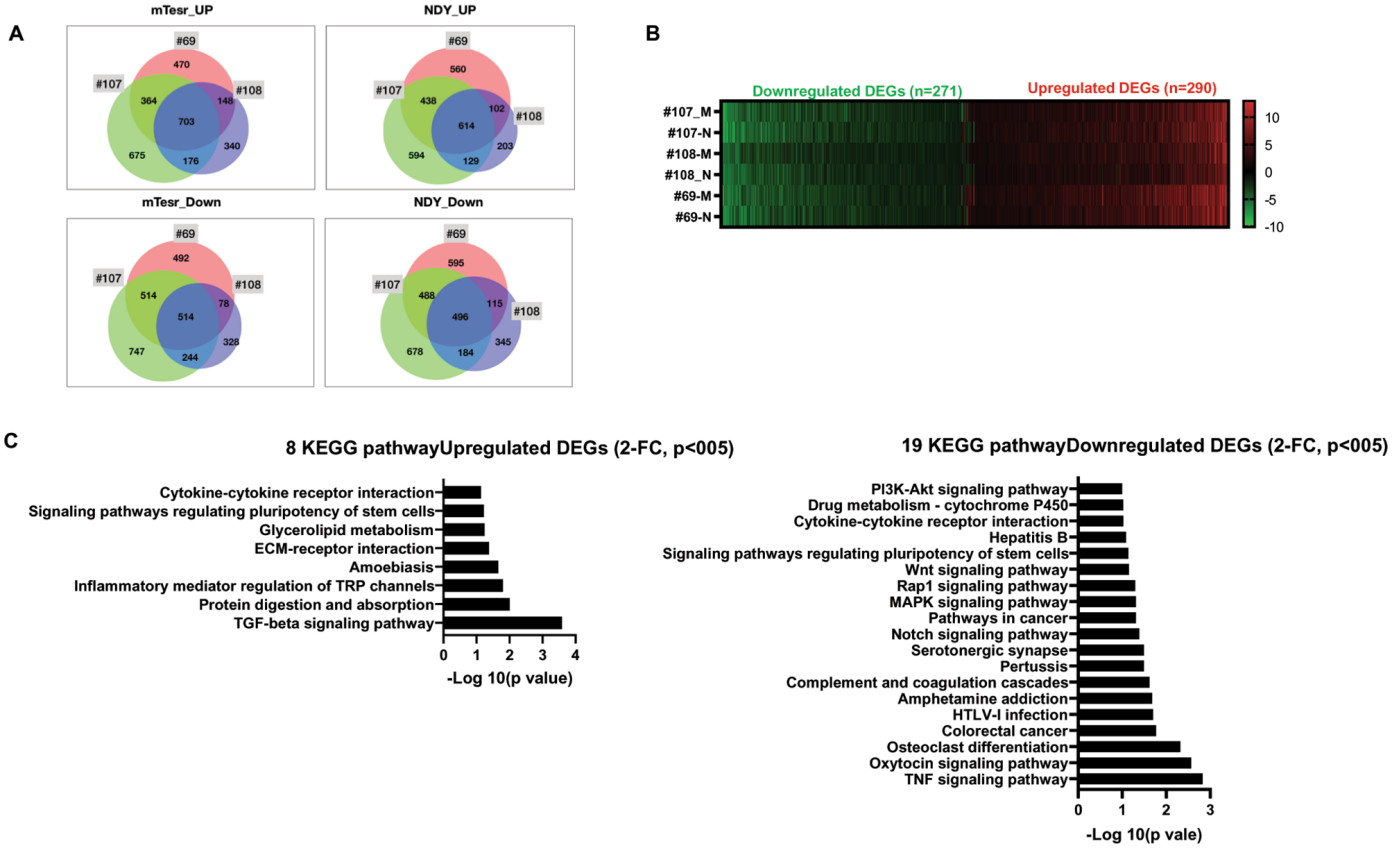
Comparison of the differentially expressed genes (DEGs) and Kyoto Encyclopedia of Genes and Genomes (KEGG) pathway enrichment between multicellular spheroids and monolayers. (**A**) Venn diagram showing a number of DEGs with 2-fold changes and p < 0.05 in spheroids verse monolayer of three cases (#69, #107, and #108). For 7–8 days, PDBCCs were grown in organoid medium as a monolayer and in two different media (mTeSR and NDY) as spheroids. (**B**) Heat map showing commonly up- or down-regulated DEGs with 2-fold changes and p < 0.05 in spheroids of three cases (#69, #107, and #108) cultivated in both media. (**C**) KEGG pathway enrichment analysis for up-and down-regulated genes between monolayers verse spheroids

Because media formulation may affect the spheroid formation, we here generated spheroids in three media systems (mTeSR, NDY, modified organoid medium). All cases of PDBCCs formed spheroids on polymer-X coated plates regardless of the three types of medium. To examine those spheroid-specific gene expression levels, we performed RNA-seq in both monolayers and spheroids and investigated up- or down-regulated DEGs (FC ≥ 2, p < 0.05) from analysis of paired-wise comparisons between each spheroid and monolayer. The total number of DEGs identified from #69 PDBCC spheroids (mTeSR; 3283, NDY; 3408), #107 PDBCC spheroids (mTeSR; 3937, NDY; 3621), and #108 PDBCC spheroids (mTeSR; 2531, NDY; 2188) was different according to the composition of the medium. The venn diagram showed the number of overlapped and non-overlapped DEGs found in 69, 107, and 108 spheroids generated in mTeSR and NDY media (Fig. 5A). The number of DEGs up-regulated in the three groups grown in mTeSR and NDY media was 703 and 614, respectively. The number of DEGs down-regulated in the three groups grown in mTeSR and NDY media was 514 and 496, respectively. We identified 561 DEGs including 290 upregulated and 271 downregulated genes that overlapped in 69, 107, and 108 spheroids regardless of the medium (Fig. 5B). The lists of top 30 up-or down-regulated genes ordered by p-value and FC in spheroids were shown in Table 3. Up- and down-regulated genes in the top rankings were identified: MMP1, MMP13, MMP10, flavin containing dimethylaniline monooxygenase 1 (FMO1), and apelin (APLN) are mainly involved in regulating extracellular matrix (ECM), cell-cell adhesion, and drug metabolism. To further understand the high-level functions and utilities of the biological system for DEGs in spheroids, KEGG pathway enrichment analyses revealed that 8 pathways including TGF-β signaling pathway, protein digestion and absorption, ECM-receptor interaction in up-regulated DEGs and 19 pathways including tumor necrosis factor (TNF), Notch, mitogen-activated protein kinase (MAPK), Rap1, Wnt, phosphatidylinositol-4,5-bisphosphate 3-kinase (PI3K)-Akt signaling pathways and drug metabolism in down-regulated DEGs were significantly enriched (Fig. 5C). A signaling pathway regulating pluripotency of stem cells was identified in both up-and down-regulated DEGs (Fig. 5C).

To identify prognostic genes related to poor prognosis among the up- or down-regulated DEGs identified from 3D spheroids, we next conducted recurrent free survival (RFS) analysis using the KM-plotter database. Clinically poor prognosis-associated 29 DEGs were selected from tumor spheroids based on RFS analysis using the Kaplan-Meier Plotter database including ER + patients (N = 3499) with a follow-up of 240 months (Table 3).

**Table 3:** Clinically poor prognosis-associated DEGs selected from tumor spheroids based on RFS analysis from the Kaplan-Meier Plotter database including ER + patients (N = 3499) with follow-up of 240 months

	Genes symbol	Log2(FC) P < 0.05					95% CI for Exp(B)		
						HR	lower	Upper	P-value
Multigene Prognostic Test Kit in Breast Cancer Patients									
Oncotype Dx	CTSV(CTSL2) CD68	5.03 4.37				1.67 1.16	1.43 0.98	1.94 1.36	2.7e-11 0.085
MammaPrint	MMP9 CDCA7L ZNF385B	6.79 -2.31 -2.57				1.4 1.56 0.66	1.19 1.15 0.48	1.65 2.1 0.92	3.9e-5 0.035 0.12
Prosigna™ (PAM50)	FOXA1 KRT17 TMEM45B	-2.28 2.59 3.16				0.85 0.71 0.71	0.73 0.61 0.53	0.99 0.83 0.94	0.042 1.3e-05 0.015
Top 30 of DEGs with 2-FC and P < 0.05									
Upregulated genes	MMP1 BMP6 MYCN COL22A1 CYP1A1(CP11) PGBD5 S100P CALML5 PPP2R2C PMEPA1	10.90 8.85 7.20 7.12 7.01 6.78 6.70 6.24 6.19 6.07				1.61 1.2 1.23 1.57 1.27 1.22 1.49 1.27 1.49 1.18	1.38 1.02 1.04 1.17 1.05 1.05 1.28 1.08 1.13 1.01	1.87 1.04 1.46 2.11 1.53 1.42 1.73 1048 1.97 1.4	7.5e-10 0.024 0.015 0.0026 0.012 0.011 3.3e-07 0.0034 0.005 0.043
Downregulated gene	FOSB LINC00844 DLK2 IL33 SCN2B CLDN19 FOS EGR1 JAM2 MYH11 ALPL	-6.36 -6.01 -5.46 -5.31 -5.09 -4.80 -4.67 -4.63 -4.62 -4.38 -4.35				0.63 0.67 0.82 0.65 0.8 0.66 0.67 0.68 0.8 0.76 0.82	0.52 0.48 0.71 0.55 0.67 0.49 0.57 0.58 0.68 0.66 0.71	0.76 0.93 0.96 0.77 0.95 0.89 0.79 0.82 0.95 0.89 0.96	2e-06 0.017 0.011 3.1e-7 0.0099 0.0065 8.5e-7 2.3e-0.5 0.0092 0.00045 0.015

B, coefficient of regression; HR, hazard ratio; CI, confidence interval; ER, estrogen receptor,; RFS, recurrent free survival; FC, fold change

## Discussion

In vitro culture of PDBCCs offers many advantages in basic and clinical breast cancer studies for shedding light on a patient’s tumor phenotype and gene expression by deciphering the effect of external factors and 2D and 3D cell-cell interaction. Recently, Sach et al. recently provided the medium composition that includes niche components for long-term growth and replication of the histological and genetic characteristics of the original tumors of PDBCCs grown as organoids [13]. Here, we employed the medium composition described by Sach et al., but without the P38 inhibitor and with 0.16 nM estradiol. The PDBCCs isolated from the fresh surgical tumor tissues of 10 patients with ER + breast cancer were successfully grown as 2D monolayers and 3D multicellular spheroids.

EGF has been identified to induce both normal and malignant epithelial cell motility and to regulate EMT and has been connected to basal breast cancer progression [19, 20]. The ALK-5 inhibitor A83-01 inhibits Smad signaling and EMT by transforming growth factor-β and becomes a target for cancer stem-like cell treatment [21]. R-spondin1 has been featured as a Wnt agonist, serving as a potent niche factor for adult stem cells in multiple tissues including the mammary gland [22, 23]. In the present study, EGF, the ALK inhibitor A83-01, R-spondin 1, and Noggin did not significantly change the growth rate of PDBCCs in 2D monolayers on collagen I-coated plates, but these factors did impact their immunophenotype; EGF removal increased the EpCAM+/CD49- mature luminal cells, R-spondin removal decreased EpCAM+/CD24 + luminal cells, and A83-01 removal reduced the EpCAM low/-/CD24-/CD44 + BCSCs. As a result, EGF and R-spondin may control the phenotypes of basal cells and luminal cells, respectively. The EpCAM low/-/CD24-/CD44 + BCSC population may be regulated by ALK-5-mediated signaling. A 3D multicellular spheroid is a useful model for enriching BCSCs and mimicking the physical interactions between solid tumors in vivo [24]. We found that the 3D multicellular spheroids of PDBCCs grown on polymer-X coated plate significantly increased basal progenitors (EpCAM-/CD49+), BCSCs (EpCAM low/-/CD24-/CD44+, ALDH high/+), and EMT-related genes as compared to the 2D monolayer, indicating that ER + PDBCCs can transform into BCSCs with strong ALDH activity and EMT characteristics when grown on polymer-X. When PDBCCs were grown as monolayers, spheroids, and organoids in the modified organoid conditions described by Sachs et al. (with 0.16 nM estradiol and no P38 inhibitor), the cell population exhibiting BCSC phenotypes was enriched in spheroids and organoids compared to monolayers. Spheroid culture in a polymer-X coated system is, in our opinion, simpler and easier than organoid culture for assessing BCSC characteristics (Supplementary Fig. 2).

DEGs identified from the transcriptomes have been utilized as a guide for molecular changes in better understanding tumor phenotypic heterogeneity and predicting prognosis [3, 25, 26]. Cell cycle-related genes are downregulated and ECM-associated genes are overexpressed, according to RNA-seq analysis of 14 breast cancer cell lines in 3D spheroids relative to 2D monolayer [27]. We found the downregulation of cell cycle and TNF signaling pathway-related genes and upregulation of ECM, cell-cell adhesion, and TGF-β signaling pathway genes in 3D spheroids relative to 2D monolayer. Online Kaplan-Meier plotters revealed that 15 up- and 14 down-regulated DEGs found in 3D spheroids relative to 2D monolayers were linked with a poor prognosis for breast cancer patients. However, artificially altering ex vivo conditions does not necessarily translate into knowledge that would ultimately aid clinical decision-making. Though DEGs show prognostic value, this is often the case when using a sufficient number of markers. To convincingly show the relevance of this study to clinical translational application, it is necessary to conduct in-depth research on both ex vivo conditions and in vivo original patient tissue.

## Conclusion

Taken together, PDBCCs derived from fresh tissues of ER + breast cancer patients successfully grew as 2D monolayers on collagen I and 3D multicellular spheroids on polymer-X film. We found that the media composition and culture method is crucial in controlling PDBCCs phenotypes.

### Electronic supplementary material

Below is the link to the electronic supplementary material.

Supplementary Material 1

Supplementary Material 2

Supplementary Material 3

## Data Availability

The RNA-sequence data from this study are deposited in the Genome Sequence Archive (GSA) (https://ngdc.cncb.ac.cn/gsa/) with GSA-Human submission ID (subHRA006164) and the BioProject accession number (PRJCA016040). The datasets used and/or analyzed during the current study are available from the corresponding author on reasonable request.

## List of abbreviations

PDBCC: patient-derived breast cancer cell
BCSC: breast cancer stem cell
ER: estrogen receptor
PR: progesterone receptor
SA-β-gal: senescence-associated beta-galactosidase
EGF: epidermal growth factor
TGF-β: transforming growth factor beta
FGF: fibroblast growth factor
ALK: activin receptor-like kinase
ALDH: aldehyde dehydrogenase
RNA-seq: RNA-sequencing
IDC: invasive ductal carcinoma
EpCAM: epithelial cellular adhesion molecule
CK: cytokeratin
KEGG: Kyoto Encyclopedia of Genes and Genomes
DEG: differentially expressed gene
RFS: relapse-free survival
EMT: epithelial -mesenchymal transition
MET: mesenchymal-epithelial transition
MMP: matrix metallopeptidase
GAPDH: glyceraldehyde-3-phosphate dehydrogenase
FMO1: dimethylaniline monoxygenase 1
APLN: apelin
ECM: extracellular matrix
TNF: tumor necrosis factor
MAPK: mitogen-activated protein kinase
PI3K: phosphatidylinositol-4,5-bisphosphate 3-kinase
