# Supplementary material for: Impact of media compositions and culture systems on the immunophenotypes of patient-derived breast cancer cells
Source: BMC Cancer. 2023 Sep 6;23:831. doi: 10.1186/s12885-023-11185-7 (PMC10481485; doi:10.1186/s12885-023-11185-7)
Supplement: Supplementary file 1 — Supplementary Material 1 [file 12885_2023_11185_MOESM1_ESM.docx]

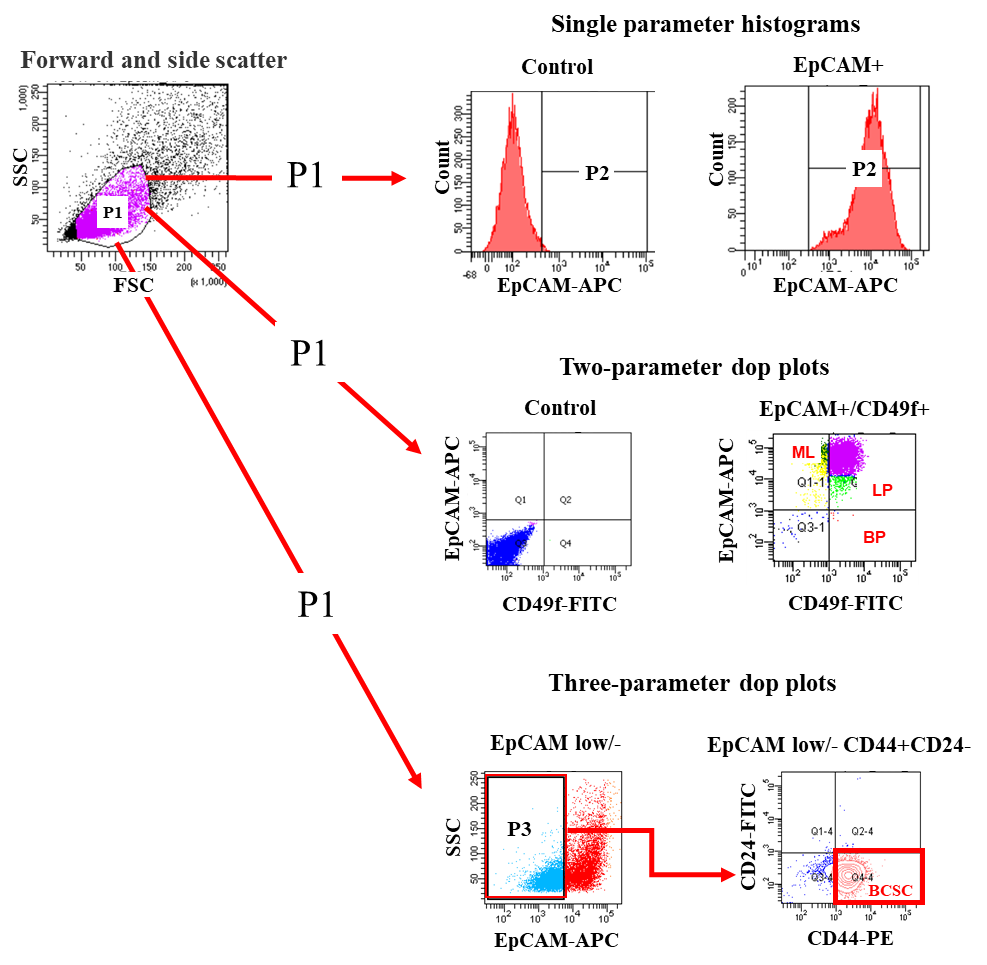
Supplementary Figure 1. The gating strategy is shown by the red arrows.


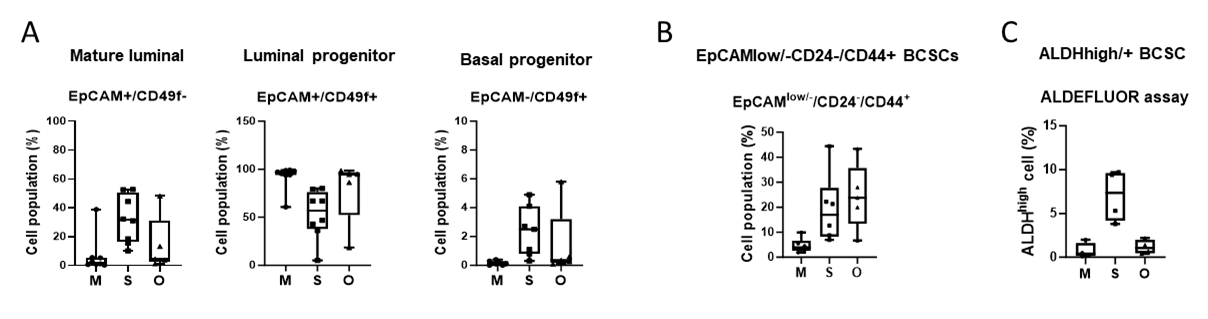
Supplementary Figure 2. Analysis of BCSCs and luminal and basal progenitors in PDBCCs grown as monolayers in modified organoids medium (M), spheroids (S), and organoids (O) using flow cytometry. (A) Analysis of EpCAM+/CD49f- mature luminal cells, EpCAM+/CD49f+ luminal progenitors, and EpCAM-/CD49f+ basal progenitors using EpCAM and CD49f double-stained flow cytometry (B) Analysis of EpCAM low/-/CD24-/CD44+ BCSCs using flow cytometry with EpCAM, CD44, and CD424 triple-stained flow cytometry (C) Analysis of ALDH high BSCSs using of ALDEFUOR™ assays
