# Supplementary figures and images for: Impact of media compositions and culture systems on the immunophenotypes of patient-derived breast cancer cells
Source: BMC Cancer. 2023 Sep 6;23:831. doi: 10.1186/s12885-023-11185-7 (PMC10481485; doi:10.1186/s12885-023-11185-7)

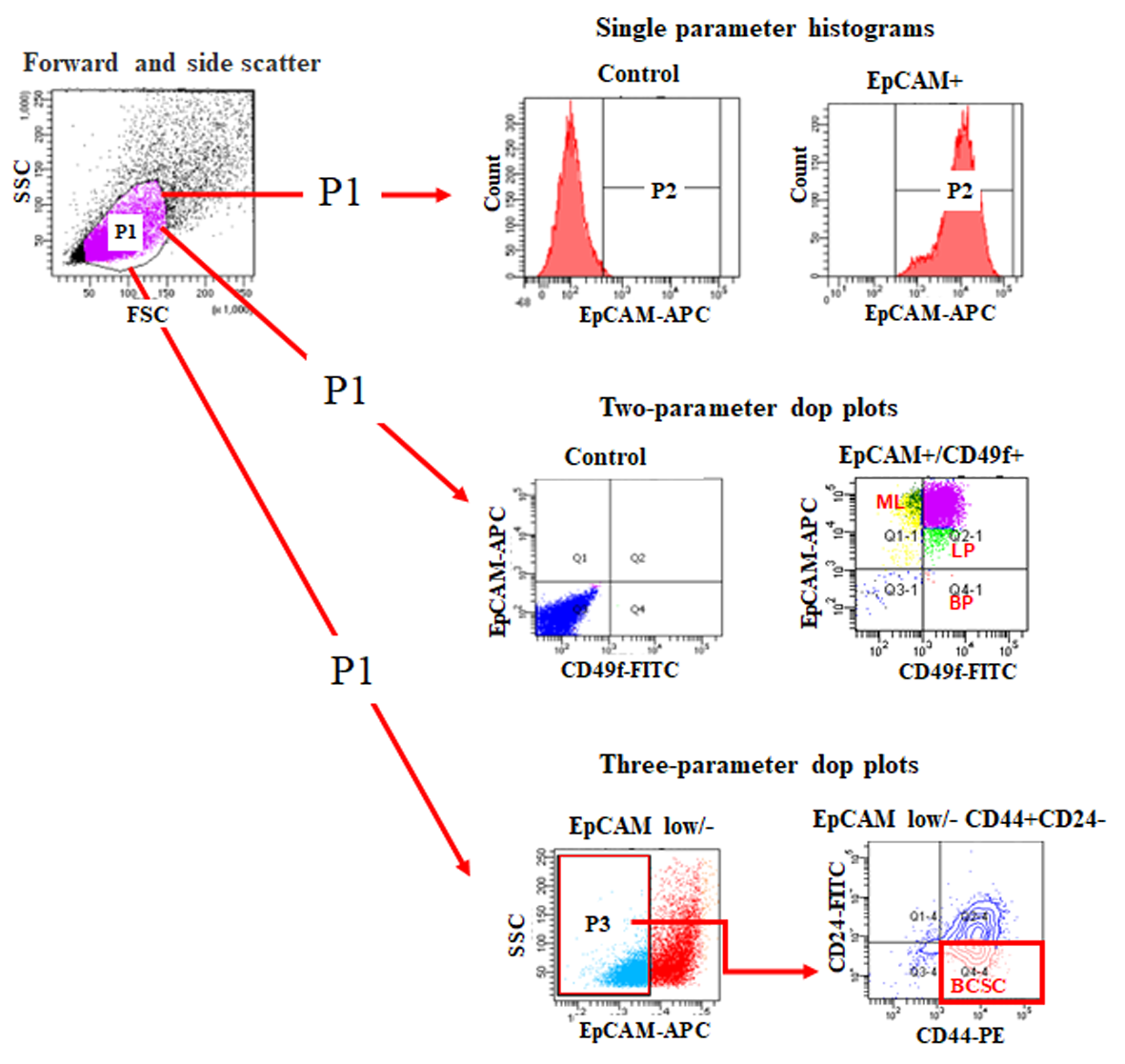

Supplement: Supplementary file 2 — Supplementary Material 2 [file 12885_2023_11185_MOESM2_ESM.png]

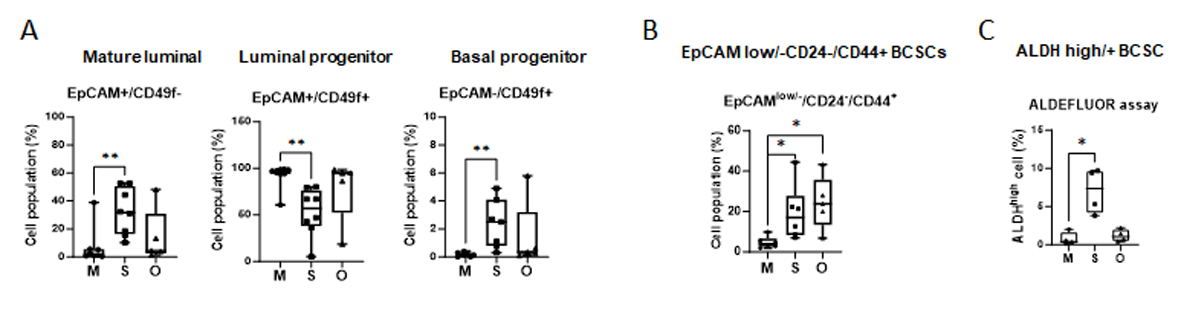

Supplement: Supplementary file 3 — Supplementary Material 3 [file 12885_2023_11185_MOESM3_ESM.png]
